# Stated Preference for Cancer Screening: A Systematic Review of the Literature, 1990–2013

**DOI:** 10.5888/pcd13.150433

**Published:** 2016-02-25

**Authors:** Carol Mansfield, Florence K. L. Tangka, Donatus U. Ekwueme, Judith Lee Smith, Gery P. Guy, Chunyu Li, A. Brett Hauber

**Affiliations:** Author Affiliations: Carol Mansfield, A. Brett Hauber, RTI Health Solutions, RTI International, Research Triangle Park, North Carolina; Donatus U. Ekwueme, Judith Lee Smith, Gery P. Guy, Jr, Chunyu Li, Centers for Disease Control and Prevention, Atlanta, Georgia.

## Abstract

**Introduction:**

Stated-preference methods provide a systematic approach to quantitatively assess the relative preferences for features of cancer screening tests. We reviewed stated-preference studies for breast, cervical, and colorectal cancer screening to identify the types of attributes included, the use of questions to assess uptake, and whether gaps exist in these areas. The goal of our review is to inform research on the design and promotion of public health programs to increase cancer screening.

**Methods:**

Using the PubMed and EconLit databases, we identified studies published in English from January 1990 through July 2013 that measured preferences for breast, cervical, and colorectal cancer screening test attributes using conjoint analysis or a discrete-choice experiment. We extracted data on study characteristics and results. We categorized studies by whether attributes evaluated included screening test, health care delivery characteristics, or both.

**Results:**

Twenty-two studies met the search criteria. Colorectal cancer was the most commonly studied cancer of the 3. Fifteen studies examined only screening test attributes (efficacy, process, test characteristics, and cost). Two studies included only health care delivery attributes (information provided, staff characteristics, waiting time, and distance to facility). Five studies examined both screening test and health care delivery attributes. Overall, cancer screening test attributes had a significant effect on a patient’s selection of a cancer screening test, and health care delivery attributes had mixed effects on choice.

**Conclusion:**

A growing number of studies examine preferences for cancer screening tests. These studies consistently find that screening test attributes, such as efficacy, process, and cost, are significant determinants of choice. Fewer studies have examined the effect of health care delivery attributes on choice, and the results from these studies are mixed. There is a need for additional studies on the barriers to cancer screening uptake, including health care delivery attributes, and the effect of education materials on preferences.

## Introduction

Screening for certain cancers may increase the identification of early-stage disease and likelihood of successful treatment and survival (1). Screening for breast, cervical, and colorectal cancer is recommended by the US Preventive Services Task Force (USPSTF) (2). Recent analysis of the 2013 National Health Interview Survey indicates that the percentages of the population screened for breast, cervical, and colorectal cancer were 72.6%, 80.7%, and 58.2%, respectively (3), below the Healthy People 2020 recommended targets of 81.1%, 93.0%, and 70.5%, respectively (4).

Research that leads to an understanding of how patients value the attributes of health care interventions is critical to the design, development, and implementation of effective programs. Incorporating patient values in the decision-making process may result in operational policies and programs that enhance the effectiveness of health care interventions by improving the uptake of and adherence to recommended preventive health care services (5).

Stated-preference (SP) methods systematically assess the relative preferences for screening tests or the features of screening tests using questions that present hypothetical trade-offs. Furthermore, SP studies can incorporate questions to assess the factors that affect reported likelihood of uptake for cancer screening (5). Previous reviews of SP studies indicate that people have identifiable preferences for the features of cancer screening tests (6–8).

This article reviews SP studies of preferences for cancer screening tests for breast, cervical, and colorectal cancer recommended by the USPSTF that were collected using conjoint analysis (CA) and discrete-choice experiments (DCEs). CA and DCEs describe tests (or other goods) using a set of attributes (features) with varying levels and allow estimation of relative preferences for different attributes. The goal of the review was to assess the types of cancer screening test attributes researchers have considered, differentiating between attributes of the screening tests themselves and attributes that capture other elements of the patient experience. We also reviewed the use of questions to determine reported likelihood of uptake. Understanding how test attributes affect reported likelihood of uptake may help improve public health programs to increase cancer screening.

## Methods

### Stated-preference techniques

Researchers have developed several approaches consistent with economic theory to measure preferences for market and nonmarket goods, interventions, and policies (5). Revealed-preference methods use information from actual behavior or purchases to infer individuals’ preferences; SP methods use surveys or experimental methods with hypothetical scenarios to elicit preferences. There are varied SP methods, including contingent value, time-trade-off, standard gamble, and other variations. The Medical Device Innovation Consortium has more information on SP methods in health care research (9).

This review focuses on CA and DCE studies, a type of SP study where the good or policy is defined by a set of attributes with varying levels (for a general discussion, see Hensher et al [10]). These surveys allow researchers to identify and quantify the relative effect of the changes in different attributes on choices. Good practice suggests that the number of attributes should be limited depending on the nature of the attributes and that researchers should make decisions about the attributes to include and exclude (5). Researchers use their research question and findings from previous studies and pretesting to select attributes that respondents find relevant. To examine reported likelihood of uptake and attributes that influence reported uptake, researchers can include a fixed alternative in the choice question, usually a reference test representing the standard of care or the option of not getting a test, or a follow-up question asking if the respondent would get the hypothetical test they selected in the choice question. CA and DCE approaches have been used for decades in the fields of marketing, transportation, environmental policy, and health care.

### Data sources and literature review strategies

Studies eligible for this systematic review met the following criteria: was a CA or DCE study; examined patient preferences for breast, colorectal, or cervical cancer screening recommended by the USPSTF; had the full-text article available in English; and was published from January 1990 through July 2013. We excluded studies that examined cancer treatment, cancer therapy, pharmaceuticals, healthy behaviors, or cancer prevention strategies not recommended by the USPSTF. We also excluded studies that included only physicians in their sample (Table 1).

**Table 1:** Inclusion and Exclusion Criteria for Studies of Conjoint Analysis Methods and Discrete-Choice Experiments, Stated Preference for Cancer Screening, Systematic Review, 1990–2013

Criterion	Inclusion	Exclusion
Population	Patients	All other populations (eg, physicians only)
Intervention	Breast, colorectal, and cervical cancer screening recommended by the US Preventive Services Task Force	Other screening, prevention, treatment, or systems interventions
Comparator	None specified	None specified
Outcomes (primary)	Attributes included in conjoint analysis or discrete-choice experiment design; use of opt-out questions	All other
Timing	January 1990 through July 2013	Before January 1990 or after July 2013
Setting	All settings	None
Study design	Conjoint analysis or discrete-choice experiment studies	All other studies
Language	English	Non-English

We used the Preferred Reporting Items for Systematic Reviews and Meta-Analyses (PRISMA) guidelines (11) to design and perform the literature review. Database searches were conducted in PubMed and EconLit. Search terms for PubMed were (“neoplasms”[mesh] or “cancer”) and (“conjoint analysis” or “conjoint analyses” or “conjoint-analysis” or “conjoint-analyses” or “discrete choice” or “discrete-choice” or “discrete ranking” or “discrete rank”). Search terms for EconLit were (“cancer”) and (“conjoint analysis” or “conjoint analyses” or “conjoint-analysis” or “conjoint-analyses” or “discrete choice” or “discrete-choice” or “discrete ranking” or “discrete rank”).

### Study selection and data extraction

We identified 157 articles, 7 of which were duplicates. We screened 150 articles for inclusion, 114 of which were eliminated. We then screened the full text of 36 articles for eligibility; 22 articles remained for inclusion in the qualitative synthesis (Figure).

**Figure:**
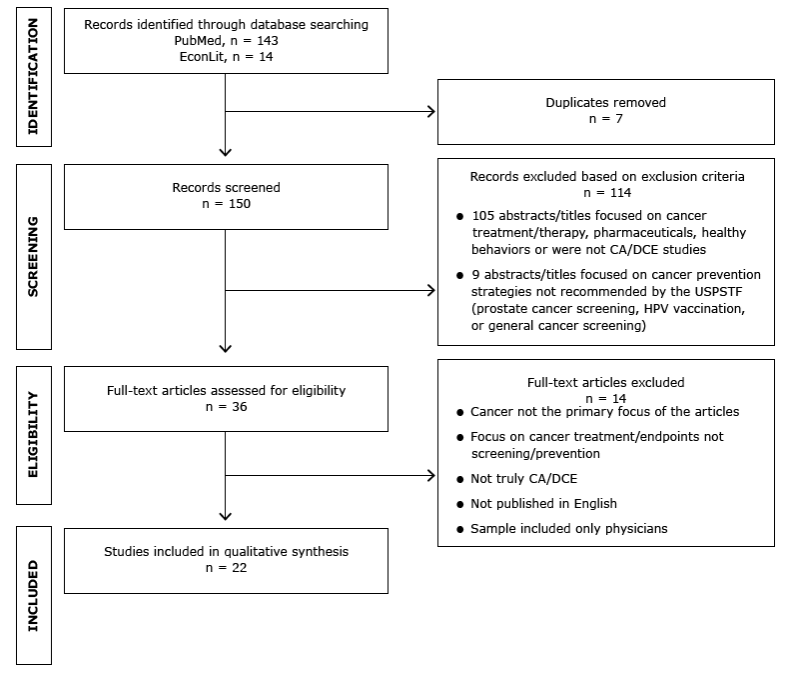
Identification and selection of articles for review. Abbreviations: CA, conjoint analysis; DCE, discrete-choice experiment; HPV, human papillomavirus; USPSTF, US Preventive Services Task Force.

We abstracted the following data items from the selected studies: author(s), year, sample size and population, cancer type, purpose of the study, attributes studied, significant attributes (defined as categorical attributes in which at least 2 levels were significantly different from each other or a continuous attribute with a significant coefficient [*P* ≤ .10]), whether the design included a no-test option, and predicted uptake as reported in the articles.

The review focused on the types of attributes included in the studies. To provide more focus for the review, the studies were categorized as studies that focused on screening test attributes only, health care delivery attributes only, or a combination of both. The categories were defined as follows:

• **Screening test attributes:** attributes of the tests independent of the patient’s characteristics. These included efficacy (sensitivity, expected reduction in cancer rates or mortality, specificity), test features (type of test, preparation before the test, length of test, pain during test, complication risk), recommended frequency, where the test was administered, how soon results were available, whether a follow-up test was needed to address abnormal findings, and cost.

• **Health care delivery attributes:** attributes related to the patient experience in the health care setting in which the screening was offered that are unrelated to the attributes of the test. These included attributes such as information provided to patients, how information was delivered, characteristics of the doctor and health care staff, waiting time for appointments, and distance to facility.

Studies were qualitatively assessed to identify common results.

## Results

Of the 22 studies, 15 included only screening test attributes, 2 included only health care delivery attributes, and 5 were a mixture of the 2. Tables 2 and 3 summarize the study characteristics and results.

**Table 2:** Characteristics of Included Studies, Stated Preference for Cancer Screening, Systematic Review, 1990–2013

Citation	Population and Sample Size	Cancer type	Purpose of Study
**Studies with only screening test attributes**			
Araña et al, 2006 (12)	60 Students in Gran Canaria, Spain (compared preferences to those of 60 oncologists)	Cervical	Compare the preferences of general population with preferences of subjects with medical expertise.
Basen-Engquist et al, 2007 (13)	Women with (n = 457) and without (n = 449) a history of abnormal Papanicolaou smear who live in Groot-Rijnmond, Netherlands	Cervical	Compare the preferences of women with and without a history of abnormal Papanicolaou smear tests, including a new technology.
de Bekker-Grob et al, 2010 (14)	Adults aged 50–74 years with (n = 649) and without (n = 626) a colorectal cancer screening history in the Netherlands	Colorectal	Compare preference results for a labeled and an unlabeled discrete choice experiment.
Gyrd-Hansen, 2000 (15)	207 Women aged 50 years living in Denmark	Breast	Assess women’s preferences for the attributes of breast cancer screening programs.
Gyrd-Hansen and Søgaard, 2001 (16)	483 Adults aged 50 years living in Denmark	Colorectal	Assess women’s preferences for the attributes of colorectal cancer screening programs.
Hawley et al, 2008 (17)	205 White, Hispanic, and African- American primary care patients aged 50–80 years with no personal or family history of colorectal cancer living in the United States	Colorectal	Describe preferences for a range of existing and new colorectal cancer screening tests among African American, Hispanic, and white primary care patients.
Hol et al, 2010 (18)	489 Screening-naive adults aged 50–74 years and 545 subjects of a colorectal cancer screening trial also aged 50–74 years living in the Netherlands	Colorectal	Assess preferences and predict the uptake of colorectal cancer screening programs and identify differences in preference structures among subgroups in the sample.
Howard and Salkeld, 2009 (19)	1,150 People who had purchased a fecal occult blood test in the past year who were living in Australia	Colorectal	Explore the effect of attribute framing on colorectal cancer screening preferences.
Howard et al, 2011 (20)	130 Patients with clinical indications suspicious of colorectal cancer who experienced both CTC and colonoscopy who are living in South Australia	Colorectal	Assess preferences of patients with suspicious clinical indications of colorectal cancer who have experienced both CTC and colonoscopy.
Marshall et al, 2007 (21)	547 Primary care patients aged 40–60 years living in Canada	Colorectal	Measure and quantify preferences for various colorectal cancer screening tests and predictors of uptake.
Marshall et al, 2009 (22)	501 General population respondents living in Canada and 1,087 living in the United States (compared with physicians)	Colorectal	Compare preferences of the general population and physicians for attributes of colorectal cancer screening tests and predictors of uptake.
Pignone et al, 2012 (23)	104 Adults aged 48–75 years with no personal or immediate family history of colon cancer, polyps, or inflammatory bowel disease living in the United States	Colorectal	Compare preferences elicited using choice-based conjoint analysis and a rating and ranking task for colorectal cancer screening tests.
Ryan and Skåtun, 2004 (24)	491 Women aged 18–65 years eligible for screening for cervical cancer and living in Scotland, United Kingdom	Cervical	Explore the importance of including an opt-out or no-test option in discrete-choice studies.
van Dam et al, 2010 (25)	152 Screening-naive individuals aged 50–74 years and 120 screening trial participants of average colorectal cancer risk living in the Netherlands	Colorectal	Compare preferences for attributes of 3 common colorectal cancer screening tests.
Wordsworth et al, 2006 (26)	577 Women aged 18–65 years eligible for screening for cervical cancer and living in Scotland, United Kingdom	Cervical	Elicit preferences for the attributes of cervical cancer screening tests.
**Studies with only health care delivery attributes**			
Griffith et al, 2009 (27)	120 Patients at high, moderate, and low risk of developing genetic cancer who received a genetic risk assessment and live in Wales, United Kingdom	Breast	Compare the preferences for attributes of genetic screening tests among women at low, moderate, and high risk of carrying a genetic mutation.
Peacock et al, 2006 (28)	339 Ashkenazi Jewish women living in Australia who enrolled in a study to test for mutations in the genes BRCA1 and BRCA2	Breast	Assess preferences for attributes of breast cancer genetic counseling services among Ashkenazi Jewish women.
**Studies with both screening test and health care delivery attributes**			
Fiebig et al, 2009 (29)	167 Women in Australia aged 18–69 years previously screened for cervical cancer (compared with general practitioners)	Cervical	Compare the preferences of consumers and providers for attributes of alternative cervical screening tests.
Gerard et al, 2003 (30)	87 Women in Australia attending breast cancer screening	Breast	Assess preferences for alternative breast cancer screening options and illustrate how breast cancer screening service providers can use empirical findings to develop preferred participation strategies.
Nayaradou et al, 2010 (31)	656 Members of the general population living in France aged 50–74 years	Colorectal	Assess preferences for different types of the fecal occult blood test, a colorectal cancer screening test.
Salkeld et al, 2000 (32)	336 People living in Australia who had used the bowel scan test kit on at least 2 occasions in the previous 3 years	Colorectal	Compare consumer preferences for an existing colorectal cancer test with a new test.
Salkeld et al, 2003 (33)	301 Adults living in Australia aged 50–70 years at “average” risk of colorectal cancer	Colorectal	Elicit preferences for attributes of colorectal cancer screening using the fecal occult blood test.

Abbreviations: BRCA1 and BRCA2, breast cancer 1 and 2, early onset genes; CTC, computed tomography colonography.

**Table 3:** Results of Included Studies, Stated Preference for Cancer Screening, Systematic Review, 1990–2013

Citation	Attributes Evaluated	Included “No Test” or Opt-Out Option?	Predicted Uptake for a Specific Test
**Studies with only screening test attributes**			
Araña et al, 2006 (12)	For practitioners, students, and pooled sample: • Interval between tests^a^ • Probability of a false-positive^a^ • Probability of dying from cervical cancer^a^ • Waiting time for the results of the test • Cost of the test^a^	Yes	Not reported
Basen-Engquist et al, 2007 (13)	• Pain^a^ • Time of results and treatment^a^ • Specificity^a^ • Sensitivity^a^	No	Not reported
de Bekker-Grob et al, 2010 (14)	• Reduction in mortality • Frequency of screening per 10 years • Complication risk • Location of screening • Screening duration • Preparation for patient • Side effects of screening	Yes	Not reported
Gyrd-Hansen, 2000 (15)	• Number of tests performed over the next 25 years • Risk of false-positive diagnosis over 30 years^a^ • Risk reduction over lifetime^a^ • Cost of the test^a^	Yes	For a program screening the 50–69-year-olds every second year, the estimated participation rates are 80.1% and 88.3%.
Gyrd-Hansen and Søgaard, 2001 (16)	• Number of tests performed over the next 25 years • Risk of false-positive diagnosis over 30 years • Risk reduction over lifetime^a^ • Cost of the test^a^	Yes	Not reported
Hawley et al, 2008 (17)	• What the test involves (eg, stool sample, X-ray)^a^ • Accuracy for finding cancer^a^ • Test frequency: how often you need to do the test^a^ • Discomfort during the test^a^ • Preparation needed for the test • Sedation^a^ • Follow-up	No	Not reported
Hol et al, 2010 (18)	• Screening test^a^ • Screening interval (years)^a^ • Risk reduction of mortality^a^	Yes	For screening-naive subjects with realistic screening intervals and mortality reduction from the literature, predicted uptake was 68% for FOBT, 79% for FS, and 77% for TC.
Howard and Salkeld, 2009 (19)	• Accuracy of test for cancers^a^ • Accuracy of test for polyps^a^ • Cost of the test^a^ • How accurate the test is at saying you do not have cancer^a^ • Preparation: dietary and medication restrictions^a^ • Process: how sample is collected^a^	No	Not reported
Howard et al, 2011 (20)	• Probability of needing a second procedure after CTC to treat polyps or cancer^a^ • Bowel preparation^a^ • Test accuracy (likelihood of missing small cancers/polyps)^a^ • Cost of the test^a^	No	Not reported
Marshall et al, 2007 (21)	• Process^a^ • Pain^a^ • Preparation^a^ • Sensitivity (accurate if you do have cancer)^a^ • Specificity (accurate it you do not have cancer)^a^ • Cost of the test^a^	Yes	The study predicted that if all colorectal cancer tests were available rather than FOBT alone then screening uptake would increase 42%.
Marshall et al, 2009 (22)	• What do I do to prepare?^a^ • Is there pain/discomfort?^a^ • How often will the screening test be done?^a^ • How is it done?^a^ • If this screening test result is abnormal, will an additional test be needed to confirm whether you have cancer? • If 10 people without cancer get this screening test, how many of them will the test say do have cancer?^a^ • If 10 people with cancer get this screening test, how many of them will the test say do not have cancer?^a^ • How many people who get this screening test have a complication?^a^ • Cost of the test^a^	Yes	Not reported
Pignone et al, 2012 (23)	• Ability to reduce colorectal incidence and mortality^b^ • Discomfort^b^ • Nature of the test^b^ • Frequency^b^ • Complications^b^ • Cost of the test^b^	No^c^	Not reported
Ryan and Skåtun, 2004 (24)	• Time between Papanicolaou smears^a^ • Waiting time for results^a^ • Chance of being recalled^a^ • Chance of abnormality^a^ • Chance of dying from cervical cancer^a^ • Cost of the test^a^	Yes	Not reported
van Dam et al, 2010 (25)	• Preparation^a^ • Location • Pain^a^ • Risk of complications^a^ • Risk of death from colorectal cancer^a^ • Interval (in the following 10 years you will undergo the test x times)^a^ • Duration^a^	Yes	Average estimated uptake of colorectal cancer screening was 56% for screening-naive individuals. If all screening tests reduced the risk of colorectal cancer–related death by 10%, uptake was estimated to be 72% for biennial FOBT screening, 46% for 5-yearly FS screening, and 22% for 10-yearly colonoscopy screening. If patients were aware of the possible risk reduction demonstrated in the literature, uptake would increase to 75% for biennial FOBT screening, 80% for 5-yearly FS screening, and 71% for 10-yearly colonoscopy screening (risk reduction of colorectal cancer–related death, respectively: 16%, 59%, and 74.5%). Results were available on how changing program characteristics affected uptake.
Wordsworth et al, 2006 (26)	• Time between Papanicolaou smears^a^ • Waiting time for results^a^ • Chance of being recalled^a^ • Chance of abnormality^a^ • Chance of dying from cervical cancer^a^ • Cost of the test^a^	Yes	Not reported
**Studies with only health care delivery attributes**			
Griffith et al, 2009 (27)	• Staff seen for counseling^a^ • Waiting time from referral to receipt of a letter confirming risk status^a^ • Distance to counseling^a^ • Duration of counseling appointments^a^ (not significant for low-risk women) • Availability of genetics testing only to high-risk women^a^ (not significant for high-risk women) • Cost of service^a^	Yes	Not reported
Peacock et al, 2006 (28)	• Risk information (information)^a^ • Giving advice about cancer surveillance (surveillance)^a^ • Preparing for genetic testing (preparation)^a^ • Assistance with decision making (direction)^a^	No	Not reported
**Studies with both screening test and health care delivery attributes**			
Fiebig et al, 2009 (29)	• Recommended screening interval^a^ • This GP is your regular GP/have never seen this GP^a^ • This GP is male/female^a^ • Time since last cervical screening test^a^ • Doctor’s recommendation^a^ • Doctor’s incentive payment • Cost of test^a^ • Chance of false-negative^a^ • Chance of false-positive^a^	Yes	Not reported
Gerard et al, 2003 (30)	• Method of inviting women for screening • Information included with invitation^a^ • Time to wait for an appointment • Choices of appointment times • Time spent traveling^a^ • How staff at the screening service relate to you • Attention paid to privacy^a^ • Time spent attending for mammogram^a^ • Time to notification of results • Level of accuracy of the screening test^a^	Yes	Not reported
Nayaradou et al, 2010 (31)	• Who proposes screening • Process^a^ • Sensitivity^a^ • Rate of unnecessary colonoscopy • Expected mortality reduction^a^ • Method of screening test result transmission • Cost of the test^a^	No	Not reported
Salkeld et al, 2000 (32)	• Dietary and medication restrictions^a^ • Whether your GP supervises the test^a^ • Notification of negative test result • Cost of the test kit^a^ • The chance of a false-positive test^a^	No	Not reported
Salkeld et al, 2003 (33)	• Benefit: sensitivity, colorectal cancer deaths prevented^a^ • Potential harm: specificity, number of false-positive–induced colonoscopies^a^ • Notification policy (of test result)^a^	No	Not reported

Abbreviations: CTC, computed tomography colonography; FOBT, fecal occult blood test; FS, flexible sigmoidoscopy; GP, general practitioner; TC, tomography colonography.

a Attribute is significant.

b Significance of attribute levels not reported.

c Included a single question after the discrete-choice experiment on screening test preference, in which respondents selected from a set of four unlabeled screening tests (designed to simulate fecal occult blood testing, sigmoidoscopy, colonoscopy, or a radiological test such as computed tomography colonography) or the option of no screening.

### Studies with only screening test attributes

Fifteen studies included only screening test attributes for breast cancer screening (15), cervical cancer screening (12,13,24,26), or colorectal cancer screening (14,16–23,25). Among the studies that examined preferences for colorectal cancer screening, 2 looked only at the fecal occult blood test (FOBT) (16,19) and 1 compared preferences for computed tomography colonography and colonoscopy (20). The rest included attributes defining a range of screening tests. Most studies surveyed the general population; however, many studies included respondents with screening experience or at higher risk of developing cancer (13,14,18–20,25).

DCE and CA studies can be set up as a forced choice, where respondents pick between tests, or they can include a no-test option where the respondent can select “no test” instead of the hypothetical options posed in the choice question. Two-thirds of the studies included a no-test option. In addition, 1 study included a separate question asking about preferences for specific unlabeled tests assigned with the characteristics of existing tests and included the option of no test (23). Four studies provided predictions of uptake for tests with specific characteristics. Gyrd-Hansen (15) found that predicted uptake for a hypothetical test screening people aged 50 to 69 years every second year with features drawn from the literature and a program in Denmark (80%–88%) was similar to estimates of actual uptake (88%). Hol et al (18) predicted a 77% uptake for colonoscopy for screening-naive respondents in his sample in the Netherlands based on what the authors defined as realistic assumptions for the attribute levels after reviewing the clinical literature. Marshall et al (21) estimated that total uptake for all types of colorectal cancer screening would be 42% at the highest if all currently available tests were offered to their sample in Canada. Van Dam et al (25) estimated uptake using risk reductions taken from the clinical literature to be 75% for biennial FOBT screening, 80% for 5-yearly flexible sigmoidoscopy screening, and 71% for 10-yearly colonoscopy screening for this sample from the Netherlands.

Another feature that distinguished the studies was whether the screening test was identified by the process or name of the procedure. This feature was most relevant for colorectal cancer screening, in which available tests range from stool samples to colonoscopies. De Bekker-Grob et al (14) compared an unlabeled design with a labeled design. Howard et al (20) used a labeled design. Four studies included an attribute that identified the type of colorectal cancer screening test by name or through the process (17,18,21,22). The rest of the studies described the tests through attributes related to efficacy and process without mentioning the type of test.

All studies included some kind of efficacy attribute. Forty percent defined efficacy as the accuracy of the test (the probability that the test found cancer or precancerous growths); the rest presented the reduction in risk of cancer mortality. The efficacy attributes were significant in every study. Forty-seven percent of the studies also included specificity (the risk of false negatives) as an attribute, which was significant in every study except one (16).

Test experience attributes included preparation before the test, discomfort during the test, waiting time for results, whether a follow-up visit was needed if results were abnormal, complication risk, duration of screening procedure, recommended test frequency, out-of-pocket cost, and type of facility where the test was conducted. The attributes that were always significant were preparation before the test (included in 47% of the studies), discomfort or pain during the test (included in 40% of the studies), waiting time for the results (included in 27% of the studies), complication risk (included in 27% of the studies), cost (included in 67% of the studies), and the type of facility where the test was preformed (included in 13% of the studies). Waiting time to get test results was not significant in 1 of the 4 times it was included (12), location of test in 1 of 2 times (14), test frequency in 2 of 11 times (15,16), and whether a follow-up test was needed to confirm abnormal results in 1 of 4 times (22).

The primary purpose of most studies was to examine preferences for screening test features; however, 3 of the studies investigated questions about DCE or CA methods. De Bekker-Grob et al (14) looked at the effect of a labeled versus unlabeled design. Pignone et al (23) compared choice-based CAs with rating and ranking. Howard and Salkeld (19) examined the effect of attribute framing (whether sensitivity and specificity were presented as cancers found or cancers missed).

### Studies with only health care delivery attributes

Only 2 studies, which looked at preferences for genetic counseling, included exclusively what we termed health care delivery attributes (27,28). Griffith et al (27) looked at preferences for genetic testing among women with a low, moderate, or high risk of breast cancer. Peacock et al (28) examined preferences for the type of information received during counseling for women at high risk of carrying the BRCA1 or BRCA2 genetic mutations, which are associated with a higher risk for breast and ovarian cancer.

The attributes in Griffith et al (27) were related to the appointment and were all significant, except whether the screening test was available only for high-risk women (versus the entire population), which was not significant to high-risk women, and the length of the appointment, which was not significant to low-risk women. The attributes in Peacock et al (28) included 4 topics that could be discussed during counseling; all were significant.

### Studies with attributes of both a screening test and health care delivery

Five studies combined screening test attributes and health care delivery attributes, and examined screening for colorectal cancer (31–33), cervical cancer (29), or breast cancer (30). Nayaradou et al (31) and Salkeld et al (32) did not include a no-test option, whereas the other studies did. Gerard et al (30) designed questions with a single scenario for screening, and women were asked if they would attend.

Nayaradou et al (31) and Salkeld et al (33) surveyed average risk or general population samples. Fiebig et al (29) compared women with and without screening histories, Gerard et al (30) sampled from women with a history of screening, and Salkeld et al (32) surveyed individuals who had used an at-home FOBT (bowel screening) kit.

Four studies included sensitivity of the screening test, reduction in cancer mortality, or both, and 4 included the chance of a false-negative (specificity). These attributes were significant in all the studies, except specificity, defined as rate of unnecessary colonoscopy in Nayaradou et al (31). Cost was included in 3 of the studies and was consistently significant (29,31,32).

The health care delivery attributes were more diverse and context specific, and many were nonsignificant. Whether a person would be notified of negative test results was significant in Salkeld et al (33) and nonsignificant in Salkeld et al (32). Whether the doctor was paid an incentive was nonsignificant in Fiebig et al (29), but other attributes related to the doctor or general practitioner were significant. Who proposed the screening or where the respondent was told they learned about the screening was nonsignificant in Gerard et al (30) and Nayaradou et al (31). Gerard et al (30) examined many features related to the appointment: some were significant (travel time to the appointment, a private changing area, and the length of the screening), and some were nonsignificant (waiting time for an appointment and the results, a choice of hours for appointments in the evening or Saturday, and whether the staff at the clinic was welcoming or reserved).

## Discussion

Overall, the studies suggest that respondents valued improvements in attributes related to the characteristics of cancer screening tests, including sensitivity, process, and cost. The significance of the health care delivery attributes was uneven across studies, especially in studies combining test and health care delivery attributes. More than half of the studies included only screening test attributes. Thirteen included some type of opt-out option, but only 4 calculated predicted uptake for specific tests.

Three similar reviews of cancer screening tests have been published. Phillips et al (6), which reviewed SP contingent valuation, CA, and DCE studies published through May 2005 for any type of cancer screening test, identified 8 studies of patient preferences. Marshall et al (7) reviewed 6 SP studies for colorectal cancer screening published between 1990 and May 2009. Ghanouni et al (8) reviewed 7 CA studies of colorectal cancer screening tests to assess the quality of the research and results. With a larger sample of 22 studies, we confirmed the findings in the earlier reviews — that patients had preferences over multiple attributes and that sensitivity was an important feature. This review included articles published through July 2013. Since this review was completed, several additional CA studies, not included in this review, have been published, including 8 more on colorectal cancer screening and 1 on breast cancer screening (34–42). Three of these more recent studies included health care delivery attributes such as travel time to breast screening appointment and the sex of staff members conducting breast screening (35,39,41). As with the 2 previous reviews (6,7), we found that most of the studies were administered to the general population at average risk of cancer; however, there are now more studies of populations at high risk of cancer or with screening histories. Several of the new studies focused on specific populations including older adults and Hispanics (34,35,39), and 1 study was conducted in Japan (41).

There are many ways in which these results from SP studies can aid in the design of future research and be applied to public health programs designed to increase screening. For example, in the United States, physicians may be more likely to recommend colonoscopy than other tests (43,44); however, the DCE and CA studies suggest that preparation, discomfort, and cost are important to patients and that some patients may prefer a stool test. In countries where stool tests are the standard of care, offering colonoscopies could improve uptake among people who have strong preferences for high sensitivity.

Health care delivery variables were sometimes nonsignificant. In SP surveys, process variables such as waiting time for an appointment may be nonsignificant relative to variables such as sensitivity, but these process factors may be important in determining whether people get screened. If an acceptable test exists, then process factors related to making appointments, getting the test, and getting the results may have a big influence on uptake for that test. Our understanding of preferences and uptake could be improved by additional research on the best way to include attributes associated with health care delivery. Health behavior theory, which has been used to develop and evaluate public health interventions (45), could provide a useful structure to develop attributes or other supporting questions related to attitude, environmental, or social factors influencing uptake (see Tsunematsu et al [41] for an example).

The hypothetical nature of SP surveys makes it challenging to accurately predict uptake. Nonetheless, adding a no-test option and providing estimates of uptake for specific tests when appropriate will provide more information on preferences and predicted uptake.

The issue of labeled versus unlabeled designs can affect predictions of uptake. De Bekker-Grob et al (14) found that choices differed based on whether labels were included. They concluded that respondents were less attentive to the attributes when labels were provided and that labeled designs may be more appropriate for respondents who were familiar with the labels and for studies interested in predicting uptake. It is unknown whether including test names as attributes is similar to using a labeled design.

We focused on patient preferences; however, studies have been done with physicians or comparing patients and physicians (12,22,29,46). Studies on physician preferences are important, because patients often rely on their physicians for advice (7). If patients and physicians value attributes differently, patient-preference surveys provide an opportunity for physicians and patients to identify differences in perspective, which could improve communication and shared decision making.

CA and DCE surveys could also be used more extensively to test the effect of messages on preferences and willingness of different populations, including underserved populations, to be screened. The results could help shape strategies for public health communication, especially because studies have found that the type of information provided can affect preferences for screening tests (7,38).

Our review has limitations. We reported attribute significance; however, the significance or lack of significance of attributes should be viewed as conditional on the set of attributes included and the range of levels. An attribute may be more or less important depending on the other attributes included in the survey. In general, best practice suggests that researchers include attributes that are important to respondents, implying that most attributes should be significant. However, even with careful pretesting, changes in attributes that are important in isolation may not be important when included in a wider set of attributes. The surveys differed in objectives and format, limiting our ability to compare findings across studies. Furthermore, few studies were conducted in the same country, which limits the generalizability of findings, because differences in national health policies vary widely among countries. For example, although many studies focused on colorectal cancer screening, only 3 were conducted in the United States.

A growing number of studies examine preferences for cancer screening tests. These studies consistently find that screening test attributes such as efficacy, process, and cost are significant determinants of choice. Fewer studies have examined the effect of health care delivery attributes on choice, and the results from these studies are mixed. Going forward, there is a need for studies on the barriers to cancer screening uptake, the impact of education materials on preferences, and the role of preference studies in patient and physician communication. Patient-preference studies may become more important as patient-centered care gains more prominence.
